# Development of Chinese mental health first aid guidelines for problem drinking: a Delphi expert consensus study

**DOI:** 10.1186/s12888-021-03266-3

**Published:** 2021-05-17

**Authors:** Wenjing Li, Anthony F. Jorm, Yan Wang, Shurong Lu, Yanling He, Nicola J. Reavley

**Affiliations:** 1 Centre for Mental Health, Melbourne School of Population and Global Health, University of Melbourne, 207 Bouverie Street, Carlton, VIC 3053 Australia; 2 Shanghai Mental Health Centre, Shanghai, China; 3 The Nossal Institute for Global Health, Melbourne School of Population and Global Health, University of Melbourne, Parkville, Victoria Australia; 4 Jiangsu Provincial Centre for Disease Control and Prevention, Nanjing, China

**Keywords:** Mental health first aid, Problem drinking, Delphi study, Mainland China

## Abstract

**Background:**

Alcohol use disorders have become the second leading cause of death for mental and substance use disorders in China. However, with early diagnosis and timely treatment, the burden can be mitigated. Family and friends of a person with alcohol use problems are well placed to recognize the signs, encourage professional help-seeking and help the person until treatment is received. We aimed to use the Delphi consensus methodology to develop guidelines about how members of the public can provide this “mental health first aid” to someone with problem drinking in China.

**Methods:**

A Chinese-language questionnaire was developed, comprising statements that were endorsed for inclusion in the English-language problem drinking first aid guidelines for high-income countries. Participants were also encouraged to suggest new statements. These statements were evaluated by two Chinese expert panels – a professional panel and a lived experience panel – on how important they believed each statement was for members of the public providing mental health first aid to a person with problem drinking in China. Three survey rounds were conducted. To be included in the final guidelines, statements had to receive a “very important” or “important” rating from at least 80% of participants from each of the panels.

**Results:**

The majority of statements were rated in the first survey round by 30 mental health professionals and 25 lived experience panel members. One hundred and eighty-one statements met the inclusion criteria and were used to form the guidelines. Compared to the English-language guidelines, the importance of family involvement and mutual support were highlighted by both Chinese expert panels, while a number of statements relating to low-risk drinking were rejected by the lived experience panel.

**Conclusions:**

The Chinese-language problem drinking first aid guidelines cover a variety of first aid strategies that members of the public can use when providing initial help to a person with problem drinking, such as how to communicate with the person and what to do if the person is intoxicated. These guidelines will be used as a stand-alone document will also inform the content of Mental Health First Aid training in China.

**Supplementary Information:**

The online version contains supplementary material available at 10.1186/s12888-021-03266-3.

## Introduction

In Chinese culture, drinking alcohol has long been accepted as an indispensable element, not only in the celebration of special events such as the Chinese New Year, but also in social networking such as building and strengthening one’s relationships with family, friends and colleagues [1–8]. While per capita alcohol consumption has declined in high-income countries in the past 30 years, it has been continuously increasing in middle-income countries such as China [9]. The 2013 Global Burden of Disease study revealed that alcohol use disorders explained 5% of mental, neurological and substance use disorder burden in mainland China, and of all mental and substance use disorders, alcohol use disorders ranked as the second leading cause of death [10, 11]. In 2016, the World Health Organization (WHO) estimated that in mainland China, 36.3% of males and 8.6% of females aged 15 years and above were heavy drinkers. Compared with 2003, the rates have been increased by approximately 27% for males and 8% for females [12, 13]. Although the estimates were much lower than the proportions reported in high-income countries at the same period such as the US (male: 41.5%; female: 11.1%) and Australia (male: 53.6%; female: 18.6%), they were much higher than other middle-income countries (e.g., 16.3% of males and 2.3% of females in Malaysia) [13].

Indeed, heavy drinking is often accepted and encouraged in a wide range of social circumstances in China, such as weddings and business dinners. In such events, repetitive toasting (i.e., encouraging a person to drink more) is a way of showing respect or sharing of joy [3, 5, 8, 14–16]. Previous research has consistently found that most of the residents in rural areas of China did not consider heavy drinking or alcohol dependence a problem, instead, being able to drink large amounts of alcohol has long been viewed as a symbol of masculinity [2, 3, 6, 7, 16–18]. Although urban residents or people with higher level of education were more likely to recognize alcohol use disorders as health issues, only a few believed that treatment was needed or had sought professional help [2, 7, 17, 18]. A recent nation-wide survey found that alcohol use was also common among mental health professionals in China, with 41.8% of 13,980 participants reporting alcohol consumption and 7.5% having alcohol use problems in the past 12 months [19]. Therefore, to improve both the public’s and healthcare professionals’ knowledge of problem drinking and their ability to help someone with alcohol use problems, a suitable and effective educational program is needed, for example, Mental Health First Aid (MHFA) training.

Similar to physical first aid, mental health first aid is often provided by members of the public (e.g., a person’s family or friends), and is defined as “the help offered to a person developing a mental health problem, experiencing a worsening of an existing mental health problem or in a mental health crisis” ([20] , p.12), until professional help is received. MHFA training courses are designed to teach the general public the skills to recognize the symptoms of different mental health problems and to provide initial help [20]. The content of the courses is based on a series of guidelines comprising a range of first aid actions that a person can undertake to help someone with a mental health problem, including alcohol use problems [21]. Evidence from recent systematic reviews and meta-analyses has shown that for both health professionals (e.g., medical students, healthcare workers) and members of the public (e.g., teachers, government employees), MHFA training is effective in improving knowledge about mental disorders, the ability to identify symptoms, willingness and confidence to provide first aid to someone with a mental health problem, and in reducing mental health stigma [22–24]. Specifically, there is evidence for the effectiveness of the training program in improving Chinese-speaking populations’ (i.e., Hong Kong Chinese and Chinese Australians) knowledge of mental health and their confidence to provide help [25, 26].

There is also evidence showing that using mental health first aid guidelines alone, which are available on the MHFA website (https://mhfa.com.au/mental-health-first-aid-guidelines), can enhance the public’s confidence and competence in providing mental health first aid, and increase both first aid providers’ and receivers’ help-seeking behaviors [21]. Although guidelines for mental health professionals on the diagnosis and treatment of alcohol use disorders have been developed in mainland China [27, 28], these guidelines are not aimed at teaching the general public how to recognize the signs of problem drinking and how to provide initial help to someone with alcohol use problems. Moreover, to our knowledge, there are no evidence-based educational interventions on problem drinking aimed at the general public in mainland China. Hence, implementing the guidelines and MHFA training in China could improve the public’s knowledge about problem drinking and their ability to provide help. However, given that the training courses and the guidelines were originally developed for high-income countries, they may not be suitable for low- and middle-income countries. Previous research, which focused on developing depression, suicide and psychosis first aid guidelines for Asian countries [29–36], has indeed found differences across cultures. For example, compared with the guidelines developed for English speaking high-income countries, first aid actions that involve a person’s family are further emphasized in the Chinese-language depression, suicide and psychosis first aid guidelines [34–36]. Therefore, the current study aimed to use the Delphi method to develop culturally appropriate mental health first aid guidelines for members of the public providing help to people with problem drinking in mainland China. For this project, problem drinking was defined as drinking behaviors that can cause short-term and/or long-term harms including physical or mental health problems, social or family difficulties, and fatal or non-fatal injuries.

## Method

This Delphi study was conducted according to the following steps as outlined by author AFJ in his article “Using the Delphi expert consensus method in mental health research” [37]: questionnaire development, recruitment of expert panel members, data collection and analyses, and guidelines development. The Delphi method was chosen because it has been demonstrated to be feasible for use in developing mental health first aid guidelines for a range of mental disorders in many countries, including Chinese-language depression, suicide and psychosis first aid guidelines [34–36], and the English-language problem drinking first aid guidelines [38].

### Questionnaire development

Our previous Delphi studies on developing Chinese-language depression, suicide and psychosis first aid guidelines have shown that statements that are included in the mental health first aid guidelines for English-speaking countries are also often endorsed by Chinese expert panels. In addition, asking panelists to suggest new helping strategies that are not mentioned in the questionnaire has been demonstrated as a feasible and efficient way to collect novel and culturally sensitive ideas [34–36]. Accordingly, statements that were endorsed for inclusion in the English-language problem drinking guidelines were included in the current questionnaire [38]. The statements, originally developed in English, were translated into Mandarin Chinese by a professional translator. The Chinese-language statements were then carefully checked and culturally tailored by the Chinese working group (WL, YH, YW and SL), comprising researchers working in the area of mental health and mental health professionals, who are not only familiar with MHFA training, but also have good knowledge of mental health treatment and health systems in China.

A total of 176 statements were included in the Round 1 questionnaire. These statements were grouped into the following 25 sections: (1) Recognizing problem drinking (4 items); (2) Understanding problem drinking (16 items); (3) Talking to the person about their drinking (23 items); (4) What to do if the person is unwilling to change their drinking behavior (8 items); (5) Professional help (7 items); (6) Discussing professional help with the person (8 items); (7) What to do if the person is unwilling to get professional help (5 items); (8) Understanding low-risk drinking (3 items); (9) How to encourage low-risk drinking (2 items); (10) Practical tips for low-risk drinking (17 items); (11) Encouraging other supports (3 items); (12) Dealing with social pressure to drink (7 items); (13) Recognizing alcohol intoxication (3 items); (14) Understanding alcohol intoxication (8 items); (15) What to do when the person is intoxicated (5 items); (16) Talking to the intoxicated person (5 items); (17) Getting the intoxicated person home (4 items); (18) What to do if the intoxicated person becomes aggressive (10 items); (19) Seeking medical help (3 items); (20) General principles for emergencies related to alcohol intoxication (6 items); (21) What to do if the intoxicated person vomits (4 items); (22) What to do if the intoxicated person falls asleep (2 items); (23) What to do if the person is dangerously intoxicated (13 items); (24) Other alcohol-related emergencies (3 items); and (25) Alcohol withdrawal (7 items). The Round 1 questionnaire also provided an open text box at the end of each section for participants to suggest helping actions and strategies that were not covered in the questionnaire. The questionnaire was administered via a Chinese online questionnaire website, Questionnaire Star.

### Expert panel recruitment

Potential participants from organizations such as Chinese Association of Drug Abuse Prevention and Treatment and Alcoholics Anonymous in China (AA) were contacted and invited by YH and YW. We used a snowball sampling method, with participants encouraged to invite others who met the following sampling criteria to join the professional expert panel or the lived experience expert panel:

1. Aged 18 and over;
2. At least secondary school education;
3. Professional expert panel – have more than two years’ working experience as a mental health professional or researcher in the area of problem drinking;
  Lived experience expert panel – have experience of problem drinking and feel well enough to join (consumers) or have caring experience for someone with problem drinking (carers).

To ensure the confidentiality and anonymity of participants, personal information, including name (or pseudonym), email and mobile number, was excluded from the exported survey data prior to analyses. Online consent was gained from each participant via selecting an “I consent to proceed” option.

### Data collection and analysis

Between December 2019 and October 2020, data were collected over three survey rounds. Participants rated each statement on a 5-point Likert scale ranging from 1 (very important) to 5 (very unimportant) according to the degree of importance for inclusion in the Chinese-language mental health first aid guidelines for problem drinking. Statements that were given “very important” or “important” ratings by at least 80% of participants from each of the panels were accepted for inclusion in the guidelines. Statements that received “very important” or “important” ratings from 70 to 79.9% of panelists from at least one expert panel were re-rated in the subsequent survey round. Statements were re-rated once only; a re-rated statement was excluded if it did not receive a “very important” or “important” rating from 80% or more of participants from both panels. If a statement received a “very important” or “important” rating from less than 70% of participants from at least one panel, it was excluded.

The suggestions for helping actions collected in Round 1 were carefully evaluated by WL, NR and YH, and new statements were generated based on those that contained novel ideas. When generating new statements, similar suggestions from multiple panel members (whether they were people with lived experience or mental health professionals) were combined and reworded. These new statements, together with statements from Round 1 that met the criteria to be re-rated, were rated in the second survey round. The third and final survey round comprised the new statements that were generated from Round 1 feedback and rated for the first time in Round 2 but needed to be re-rated in a further round. Each participant was sent a summary of Round 1 and Round 2 responses including the number of endorsed and rejected statements, and a list of statements that needed to be re-rated in the next round, together with each panel’s previous ratings for the re-rated statements.

### Guidelines development

The first author wrote the statements that were endorsed across the three rounds of questionnaires into a guidelines document. The Chinese working group then met and reviewed this document and finalized the structure and wording. The final draft of the guidelines was also sent to panelists for comments. The final guidelines can be found in online Supplementary material 1.

### Ethics approval

The study was approved by the University of Melbourne Human Ethnics Committee (HREC No.1750853.1) and the Shanghai Mental Health Center Human Ethics Committee (IRB No.2018–62).

## Results

### Round 1

A total of 55 participants completed the Round 1 questionnaire. The professional panel (*N* = 30) comprised 18 psychiatrists and 12 mental health nurses. Most of the panelists were from Shanghai (*N* = 16), 4 were from Shandong, 3 were from Yunnan, 2 were from Hunan, and 1 each from Guangdong, Heilongjiang, Liaoning, Sichuan and Xinjiang. The average age was 42.2 years (*SD* = 10.3, range 27–67), with 40% males (*N* = 12) and 60% females (*N* = 18).

The lived experience panel (*N* = 25) comprised 18 consumers and 7 non-mental health professionals who had experiences of caring for someone with problem drinking. The average age was 44.1 years (*SD* = 10.8, range 32–67), with 48% males (*N* = 12) and 52% females (*N* = 13). Six panelists were from Anhui, 5 were from Shanghai, 2 each from Guangdong and Sichuan, and 1 each from Beijing, Hebei, Heilongjiang, Hubei, Jiangxi, Liaoning, Inner Mongolia, Qinghai, Tianjin and Yunnan.

**Fig. 1:**
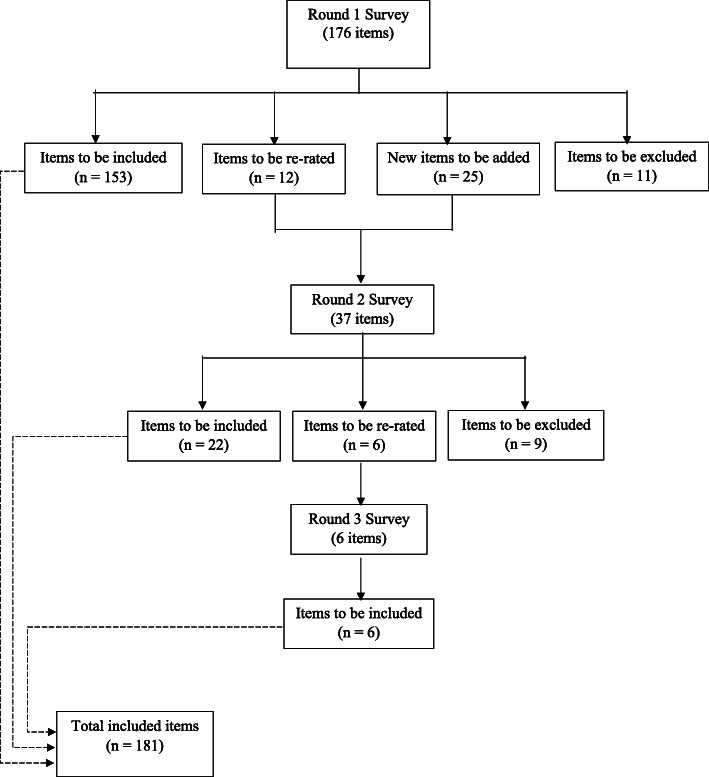
The number of items that were included, re-rated and excluded at each round of the survey

Out of the 176 statements rated in the Round 1 questionnaire, 153 statements were endorsed for inclusion in the final guidelines, with 11 rejected and 12 requiring re-rating in the Round 2 questionnaire (Fig. 1). A list of the endorsed, re-rated and rejected statements can be found in online Supplementary material 2. The endorsement rates from the two panels were significantly correlated (*r* = .58, *p* < .001). In accordance with previous research [34, 39], the endorsement ratings were compared across the professional panel and the lived experience panel. The statements that were rejected by one panel but endorsed by the other with a difference of ≥10% on the ratings are shown in Table 1. Substantial differences were noticed in 6 items relating to low-risk drinking. As described above, participants were also encouraged to suggest new helping actions in Round 1, and a total of 25 new statements were developed from both panels’ feedback.

**Table 1:** Items with notable differences on the endorsement ratings between the professional panel and the lived experience panel

	Endorsement rates	
Items	Professional	Lived experience
*Round 1*		
*Section 8: Understanding low-risk drinking*		
The first aider should be aware that abstinence from drinking may not be the person’s goal and that reducing the quantity of alcohol consumed is a worthwhile objective.	83%	68%
*Section 10: Practical tips for low-risk drinking*		
The first aider should ask the person if they would like some tips on low-risk drinking.	87%	68%
If the person wants to change their drinking behavior, the first aider should suggest tips for low-risk drinking.	83%	68%
If the person wants some advice on low-risk drinking, the first aider should advise the person not to let people top up their drink before it is finished, so they don’t lose track of how much alcohol they have consumed.	80%	60%
The first aider should advise the person to be aware of the alcohol content of their drink.	83%	64%
If the person wants some advice on low-risk drinking, the first aider should advise the person to reduce the amount of alcohol they drink by consuming drinks with lower alcohol content (for example, drinking light beer instead of full-strength beer).	83%	56%
*Round 2*		
*Section 7: What to do if the person is unwilling to get professional help*		
(re-rate) If the person is unwilling to get professional help, because they don’t want to stop drinking completely, the first aider should explain that the treatment goal may be to reduce alcohol consumption rather than to quit altogether.	90%	59%
*Section 10: Practical tips for low-risk drinking*		
(re-rate) If the person wants some advice on low-risk drinking, the first aider should advise the person to be aware of the number of standard drinks they consume.	90%	65%
*Section 11: Encouraging other supports*		
(new) The first aider should encourage the person to stop drinking completely.	55%	82%

*Note.* items with notable differences are those which were rejected by one panel but endorsed by the other, and with a difference of ≥10% on the endorsement ratings

### Round 2

The Round 2 questionnaire included the 25 new statements and the 12 needing to be re-rated (Fig. 1). A total of 29 professionals (Male = 37.9%; *M*_age_ = 42.5, *SD* = 10.4, range 27–67) and 17 lived experience panelists (Male = 47.1%; *M*_age_ = 44.7, *SD* = 10.1, range 35–66) completed the questionnaire. Twenty-two statements were endorsed, 9 were rejected, and 6 needed to be re-rated in a further round. As shown in Table 1, notable differences between the two expert panels were found in 3 items relating to professional help, low-risk drinking, and other supports, respectively.

It is worth noting that, nineteen items, which were endorsed and included in the English-language guidelines [38], were rejected by Chinese panels:

- What to do if the person is unwilling to get professional help:
  - ○ If the person is unwilling to get professional help, because they don’t want to stop drinking completely, the first aider should explain that the treatment goal may be to reduce alcohol consumption rather than to quit altogether.
- Understanding low-risk drinking
  - ○ The first aider should be aware that abstinence from drinking may not the person’s goal and that reducing the quantity of alcohol consumed is a worthwhile objective.
  - ○ The first aider should be familiar with national guidelines for low-risk alcohol consumption.
  - ○ The first aider should know what a standard drink is.
- Practical tips for low-risk drinking
  - ○ The first aider should ask the person if they would like some tips on low-risk drinking.
  - ○ If the person wants to change their drinking behavior, the first aider should suggest tips for low-risk drinking.
  - ○ If the person wants some advice on low-risk drinking, the first aider should tell the person where they can get information about low-risk drinking.
  - ○ If the person wants some advice on low-risk drinking, the first aider should advise the person to be aware of the number of standard drinks they consume.
  - ○ If the person wants some advice on low-risk drinking, the first aider should advise the person not to let people top up their drink before it is finished, so they don’t low track of how much alcohol they have consumed.
  - ○ If the person wants some advice on low-risk drinking, the first aider should advise the person to eat while drinking.
  - ○ If the person wants some advice on low-risk drinking, the first aider should advise the person to drink plenty of water on a drinking occasion to prevent dehydration.
  - ○ The first aider should advise the person to be aware of the alcohol content of their drink.
  - ○ If the person wants some advice on low-risk drinking, the first aider should advise the person to reduce the amount of alcohol they drink by consuming drinks with lower alcohol content (for example, drinking light beer instead of full strength beer).
  - ○ If the person wants some advice on low-risk drinking, the first aider should advise the person to switch to non-alcoholic drinks when they start to feel the effects of alcohol.
  - ○ If the person wants some advice on low-risk drinking, the first aider should advise the person to drink slowly, for example, by taking sips instead of gulps and putting their drink down between sips.
  - ○ If the person wants some advice on low-risk drinking, the first aider should advise the person to have one drink at a time.
  - ○ If the person wants some advice on low-risk drinking, the first aider should advise the person to think of drinking alcohol as complementary to another activity instead of the sole activity.
- Dealing with social pressure to drink
  - ○ The first aider should be aware that there is often social pressure to get drunk when drinking.
- Understanding alcohol intoxication
  - ○ The first aider should be aware that only time will reverse the effects of intoxication.

### Round 3

A total of 19 professionals (Male = 31.6%; *M*_age_ = 43.7, *SD* = 10.7, range 27–67) and 11 lived experience panelists (Male = 45.5%; *M*_age_ = 45.6, *SD* = 11.2, range 36–66) completed the third and final round of the questionnaire. All of the 6 statements, which were generated from panelists’ feedback in Round 1 and rated for the first time in Round 2 but needing to be re-rated, were endorsed, giving a sum total of 181 statements that formed the basis of the Chinese-language problem drinking first aid guidelines.

## Discussion

This study aimed to develop guidelines for members of the public providing mental health first aid to a person  with problem drinking in China. A total of 181 statements that were endorsed by both professional and lived experience panels were used to form the guidelines. These guidelines cover a variety of mental health first aid strategies, including recognizing and understanding problem drinking and alcohol intoxication, communicating with the person, encouraging low-risk drinking, professional help and other supports, dealing with social pressure to drink, emergencies related to alcohol intoxication and alcohol withdrawal, what to do if the person is intoxicated, vomits, falls asleep, or becomes aggressive because of being intoxicated, what to do if the person is unwilling to change their drinking behavior or unwilling to get professional help, and seeking medical help.

Differences between the professional and the lived experience panels were identified in endorsement ratings for several statements relating to low-risk drinking. Although these statements (e.g., “If the person wants some advice on low-risk drinking, the first aider should advise the person to be aware of the number of standard drinks they consume”) were endorsed by mental health professionals, they were rejected by members of the lived experience panel. It is possible that, unlike professionals, who understand the benefits of low-risk drinking in reducing the harmful effects of alcohol well, the lived experience panel may be worried that suggesting low-risk drinking may make the person believe that they are authorized to drink [40]. Indeed, lived experience panel members commented that low-risk drinking would become an excuse for people with alcohol use problems to drink. One lived experience panel member said, “For people with alcohol use problems, they can’t stop once they start drinking, therefore, I don’t recommend low-risk drinking …” . And another said, “… even for those who have achieved alcohol abstinence, they can return to uncontrolled drinking immediately after a sip, therefore, I don’t think low-risk drinking is a good idea”.

Moreover, some members of the lived experience panel were recruited through AA in China. As the primary purpose of AA is to stop drinking completely and stay sober (*“保持滴酒不沾”*) (http://www.aa-china.org/), which contradicts the low-risk drinking recommendations, those lived experience panel members who were recruited from AA may have disagreed with the statements relating to low-risk drinking and given lower endorsement ratings. Furthermore, it is noteworthy that of the 19 statements, which were included in the English-language problem drinking first aid guidelines but rejected by Chinese expert panels, 16 were about low-risk drinking. Unlike Australia and other English-speaking high-income countries, there are no national guidelines for alcohol use in China, therefore, lack of knowledge and awareness of low-risking drinking may be common in Chinese populations. Future research would benefit from exploring how different Chinese populations (e.g., patients, caregivers) understand low-risk drinking, including their beliefs, attitudes and knowledge. The central government could consider providing funds to develop national guidelines for alcohol intake (e.g., low-risk drinking guidelines) to improve the Chinese public’s understanding of alcohol use.

In contrast to their English-speaking counterparts, the importance of family involvement and mutual support were emphasized by Chinese expert panels. Both professional and lived experience panel members not only gave higher endorsement ratings to original first aid actions that involve the person’s family members and non-professional supports, such as “The first aider should encourage the person to spend time with non-drinking family and friends who support the person’s effort to change their drinking behavior”, “The first aider should be aware that it is beneficial for a friend or family member to accompany the intoxicated person to hospital because they may be able to provide relevant information”, and “The first aider should make the person aware of the range of non-professional supports available for problem drinking, for example, self-help groups”, but also suggested several new relevant strategies including “If the first aider is not a family member, they should provide guidance about alcohol problems to the family”, “If the intoxicated person falls asleep, the first aider should inform the person’s family”, and “The first aider should consider recommending the person to a mutual support group or take part in group therapy”. Indeed, there is evidence suggesting that family involvement and attending mutual support groups (e.g., AA) can help a person recover from an alcohol use problem and enhance treatment outcomes [41–44].

The current findings must be interpreted in the context of a few limitations we encountered. First, despite our best efforts to recruit the recommended number for each panel (i.e., 20 or more members) [37], there was a high dropout rate. Only 44% of lived experience panel members and 63% of mental health professionals participated in all three rounds. It may be because the time commitment required for the Round 1 questionnaire (i.e., approximately 1 h) deterred participants from taking part in subsequent rounds. However, the minimum panel size was achieved for each panel in the first round of questionnaire where the majority of statements were rated. Second, it is very difficult to recruit some types of mental health professionals, such as clinical psychologists, due to the lack of these professionals in mainland China [45, 46]. Despite approaching a number of organizations, we were also unable to recruit enough consumers and carers to have two separate expert panels. Instead, participating consumers and carers were combined into one lived experience panel. In addition, as mentioned earlier, some of the lived experience panelists were recruited from AA China, their experience within AA may have influenced their views on some of the statements, such as those relating to low-risk drinking. Future research would benefit from having larger and more diverse samples. The recruitment difficulty we encountered may partly be due to the public’s lack of awareness of problem drinking and relevant treatments as well as a lack of consumer advocacy organizations with a focus on problem drinking. Indeed, research conducted in China has consistently identified that people with alcohol use problems rarely seek professional help until they have developed physical health problems or severe psychiatric symptoms [7, 17].

## Conclusion

Using the Delphi consensus method, we developed culturally appropriate mental health first aid guidelines for members of the public helping people with alcohol problems in China. These guidelines provide a range of mental health first aid strategies, such as how to communicate with a person with problem drinking, what to do if the person is intoxicated, and how to deal with emergencies related to alcohol intoxication and alcohol withdrawal. Improving the public’s knowledge of mental health has consistently been mentioned in China’s mental health policies, such as the two National Mental Health Plans [47, 48]. The current guidelines will contribute significantly to enhancing the Chinese public’s understanding of alcohol use and how to provide initial help to someone with problem drinking. These guidelines can be used as a stand-alone document by the Chinese public seeking guidance when providing initial help to a person with problem drinking. The guidelines will also be used to inform the content of MHFA training for the public in China. Further research will be carried out to investigate the effectiveness of the guidelines and the training courses in improving the Chinese public’s mental health literacy and helping behaviors.

## Supplementary Information

**Additional file 1.**

**Additional file 2.**

## Data Availability

The dataset used and analyzed during the current study are available from the corresponding author on reasonable request.

## Abbreviations

MHFA: Mental Health First Aid
WHO: World Health Organization
AA: Alcoholics Anonymous
