# Supplementary material for: Development of Chinese mental health first aid guidelines for problem drinking: a Delphi expert consensus study
Source: BMC Psychiatry. 2021 May 17;21:254. doi: 10.1186/s12888-021-03266-3 (PMC8127318; doi:10.1186/s12888-021-03266-3)
Supplement: Supplementary file 1 — Additional file 1. [file 12888_2021_3266_MOESM1_ESM.docx]

# 精神健康急救指南 – 问题饮酒篇

## 指南的目的

指南旨在指导公众如何为可能正在经历问题饮酒的人（在本指南中称为“救助对象”或“对方”）提供初步帮助，即实施“精神健康急救”。

| 精神健康急救 (Mental Health First Aid, MHFA)：为发生精神健康问题，或现有精神健康问题恶化，或正在经历精神健康危机(如自杀或创伤经历)的人提供的**初步帮助**，直至对方获得适当的专业帮助或危机解除。 |
| --- |

所谓精神健康急救人员（后简称“急救人员”），即为经历问题饮酒的人提供救助的非精神卫生专业人士，他们可以是救助对象的家人、朋友、同事或邻居，等等。急救人员的作用是为经历问题饮酒的人提供初步帮助，直至对方获得适当的专业帮助或危机解除。

## 指南的制定

指南的内容是在综合经验丰富的精神健康领域专家、有照顾问题饮酒者经验人士以及有饮酒问题者意见的基础上制定的。

## 指南的使用

指南所提供的指导方针仅为一般性建议。每个救助对象的情况都是不同的，急救人员需要根据对方的情况对所提供的帮助作出适当调整。

## 识别、了解问题饮酒

你应该了解嗜酒是一种精神障碍，饮酒可导致或恶化心理健康问题。作为急救人员，你要能够识别问题饮酒、高风险饮酒的信号、酗酒及酒精依赖的症状；了解长期、大量饮酒的潜在危害以及救助对象过度饮酒的原因，例如，未经治疗的精神疾病，或把饮酒作为处理生活中问题的一种方式（如潜在的情绪困扰或精神疾病）。询问救助对象受饮酒问题困扰的时间以及饮酒量，并尝试查明他是否有酒精依赖等问题。

虽然救助对象本人是唯一可以决定改变自己饮酒行为的人，但是仅靠个人的毅力和决心有时候还不足以帮助他停止问题饮酒。你要知道摆脱依赖只是康复过程的一部分，他还需要改变许多生活习惯以改变不良饮酒行为。改变饮酒习惯是不容易的，但是有可能的，救助对象可能会反复一次或多次才会改变自己的饮酒模式。你应协助他改变不良饮酒习惯，但是只给建议并不能有效地帮助他改变饮酒行为。通常你要先解决他潜在的情绪困扰或心理健康问题才有可能终止其问题饮酒。即使他不再喝酒，你也仍应继续密切留意，直至其躯体戒断症状消失。

| **问题饮酒**是指会导致短期或长期危害的饮酒行为。这些危害包括：   - 家庭或社交困难（如人际关系、工作、经济问题） - 非致命和致命伤害（如意外事故、跌倒、暴力、交通事故） - 心理健康问题（如焦虑、抑郁） - 身体健康问题（如恶心、头痛、胃溃疡、肝脏或心脏疾病）   **高风险饮酒：**我国膳食指南建议成人饮酒量，男性应≤25g/d，女性应≤15g/d^1^。饮酒中含有酒精10g称为“一杯”，例如250ml啤酒、一小盅（15ml）烈酒、一杯葡萄酒或黄酒。超出该量通常认为是高风险饮酒。  **酒精使用障碍**是指在12个月内出现以下2个或以上情况^2^：   1. 酒精的摄入常常比意图的量更大或时间更长 2. 主观上一直有减少或控制酒精使用的愿望，但努力失败 3. 大量的时间花在那些获得酒精、使用酒精或从酒精的效果中恢复的必要活动上 4. 对使用酒精有渴求或强烈欲望 5. 反复的酒精使用导致不能履行在工作、学习或家庭生活中的角色义务 6. 尽管酒精使用已引起或加重持久的或反复的社会和人际交往问题，但仍然继续使用酒精 7. 由于酒精使用而放弃或减少重要的社交、职业或娱乐活动 8. 在对躯体有害的情况下，反复使用酒精 9. 尽管认识到使用酒精可能会引起或加重持久的、反复的生理与心理问题，但仍然继续使用酒精 10. 耐受，通过下列2项之一来定义： 11. 需要显著增加酒精的量以达到过瘾或预期的效果 12. 继续使用同量的酒精会显著降低效果 13. 戒断，表现为下列2项之一： 14. 酒精戒断综合征的特征 15. 酒精（或密切相关的物质，如苯二氮䓬类）用于缓解或避免戒断症状   ^1^ *摘自中国居民膳食指南（2016）*[*http://dg.cnsoc.org/index.html*](http://dg.cnsoc.org/index.html)  ^2^ *摘自美国精神医学学会（2013）精神障碍诊断与统计手册（DSM-5）* |
| --- |

## 与问题饮酒者谈论其饮酒问题

在开始交谈时，你可以先寻找一些共同话题与对方进行讨论，然后逐步提出他正在经历的具体问题。你也可以通过询问他生活中不良饮酒行为已造成的影响（如自身的情绪、工作表现和人际关系）来确定他是否已准备好谈论其饮酒问题。你应该试着去理解他对自己饮酒问题的看法，开诚布公地与他讨论其饮酒问题，询问他的饮酒行为（如一般喝多少酒）以及是否认为自己的饮酒行为已成为问题。鼓励他寻找关于如何减少问题饮酒危害的信息。忠告他，酒精可能会与其他药物（如违禁药或处方药）产生不可预测的不良反应，而这可能会成为紧急医疗情况。

选择在安静、私密且无其他干扰的环境中与对方交谈，并确保双方都清醒且心情平静。以支持的方式与他互动，使用含“我”的句子（如“我很担心，你最近喝了好多酒”），切忌使用威胁或咄咄逼人的语言方式，也不要向其说教。不带评判地倾听对方讲话，避免对其饮酒行为做道德评判。识别并针对其行为进行讨论，不要对其人品进行批评,如可以说“喝酒好像妨碍了你和朋友的关系”，而不说类似“你真是一个可怜的酒鬼”之类的话；也不要给对方贴“标签”（如“酒鬼”或“有酒瘾”）。

告诉对方你愿意并能够为他提供什么样的帮助，比如，愿意做一个好的倾听者，或者可以安排他接受专业帮助。不要奢望他的思想或行为会立刻发生变化，因为他可能通过此次对话才第一次意识到自己的饮酒行为是一个问题。他可能并不记得喝醉时发生的事情（即可能已昏睡），也可能没有意识到甚至压根就否认自己有饮酒问题。强迫他承认自己存在饮酒问题可能会引起双方冲突。

## 如果问题饮酒者不愿意改变饮酒行为怎么办

如果对方不愿意改变饮酒行为，你不应该：

- 与其一起喝酒；
- 遮掩或为他找借口；
- 试图通过贿赂、唠叨、威胁或哭泣来控制他；
- 替他承担责任，除非不这样做会造成伤害，如危害他自己或他人安全。

你可以尝试了解他不愿改变饮酒行为的原因，咨询专业人员以确定如何用最好的方式与其沟通，让他了解你的担忧，或者咨询那些有处理问题饮酒经验的人以寻求有效的方式来帮助他。你也可以与问题饮酒者讨论其饮酒行为可能会导致的不良后果。注意，对方可能会反复复饮，甚至有恶意攻击的可能，需要你长期的支持或帮助。如果他不愿意改变其饮酒行为，你不应觉得内疚或要为其饮酒行为负责。

## 专业帮助

救助对象可能需要专业帮助，如果他：

- 承认常常想喝酒并想着下次什么时候有机会再喝酒；
- 因花大量的钱在饮酒上而负债；
- 没有酒喝时会变得焦虑；
- 需要借助酒精来应对某些情况；
- 因饮酒导致与别人争吵或发生意外事故；
- 处理日常事务的能力受到严重干扰。

注意：除非在某些特殊情况下，例如，发生暴力事件导致警方介入或紧急医疗情况，否则不能强迫对方接受专业治疗。

## 与问题饮酒者讨论专业帮助

初次听到专业帮助时，对方可能会显得抗拒，或者可能会觉得很难接受。你应该告诉他寻求专业帮助可以使情况变好，向其传达希望。你也应该告诉他，自己会协助其获得专业帮助，并让他放心，在寻求专业帮助时个人信息是保密的。注意，对方拥有是否寻求专业帮助的最终决定权。如果他愿意寻求专业帮助，你应该给他提供当地现有服务及专业机构的信息，并应鼓励其先预约。

## 如果问题饮酒者不愿寻求专业帮助怎么办

如果对方不愿意寻求专业帮助，你应该向他解释专业帮助的益处, 或者，向他解释，有多种方法可以解决饮酒问题。同时，做好以后跟他再交谈的准备，富有同情心和耐心地等待他同意接受专业帮助。如果他正在逐步将自己或他人置于危险之中，你则应该持续建议他寻求专业帮助。设定行为界限，自己愿意或不愿意接受他哪些行为。

## 避免高风险饮酒

你应该建议对方识别其最有可能喝酒的情境，并尽可能避开。如果他需要如何少饮酒的建议，你应该：

- 告诉他一标准杯是多少（饮酒中含有酒精10g称为“一杯”，例如250ml啤酒、一小盅（15ml）烈酒、一杯葡萄酒或黄酒）；
- 建议他不要参加饮酒比赛和饮酒游戏；

建议他把时间花在不涉及饮酒的活动上。

你应该告诉他，只有他自己才能承担起减少饮酒量的责任，虽然改变饮酒模式比较困难，但不应放弃努力。

## 鼓励其他支持方式

你应该让对方了解，针对问题饮酒有各种非专业的帮助形式，如自助小组。你可以推荐他参加互助组织或团体治疗，或建议他的家人一起参加团体治疗。

你应意识到家人及其他社会支持对改变救助对象不良饮酒习惯的重要性，鼓励对方多联系支持其改变饮酒行为的朋友和家人，特别是多与那些不喝酒并能为他提供支持的朋友和家人相处。同时，提醒他，并非所有的家人和朋友都会支持其努力改变饮酒行为。如果你不是救助对象的家人则应向其家人提供问题饮酒相关知识。

## 应对饮酒的社交压力

你应该鼓励对方尽可能减少会涉及大量饮酒的应酬活动。建议他被劝酒而自己又不想多喝时，要坚决拒绝。告诉他，被劝酒时，他有权拒绝，可以说“不了，谢谢”，并且不需要作解释。你应该向他提供婉拒劝酒的不同方法，例如，“我不在状态”、“我感觉不太舒服”或“我正在吃药”。鼓励他练习面对劝酒压力时婉拒的不同方法。告诉他，真正在乎他的人会接受他不喝酒的决定。你也应该告诉他喝酒不是促进社会交往的唯一方法。

## 识别醉酒

你要能够识别醉酒的症状；知道有些疾病的症状可能与醉酒的症状相似。你也要了解导致醉酒不同症状的主要因素，如既往饮酒史、药物使用情况和身体状况。

| **醉酒**指血液中的酒精含量显著升高，严重损害人的思考能力及行为。一个人喝醉时可能会从事各种危险活动，如不安全的性行为、破坏财产或醉酒驾驶。  醉酒的症状包括：   - 口齿不清 - 动作不协调 - 走路摇晃或跌倒 - 吵闹、好辩或出现攻击性行为 - 呕吐 - 困倦或嗜睡 - 注意力或记忆损害 |
| --- |

## 了解醉酒

你要知道人体一个小时只能代谢约一标准杯的酒，喝浓茶、黑咖啡、睡觉、走路及洗冷水澡并不能解酒。醉酒可能会导致紧急医疗情况。如果你认为对方已处于危险状态，应安排其去医院。值得注意的是，他可能没有意识到自己醉得有多厉害。

## 当问题饮酒者喝醉时该怎么办

当对方喝醉时，你应该：

- 保持镇定；
- 评估当前的状况是否存在危险，并确保对方、自己及其他人的安全；
- 询问他是否正在服用任何药物或违禁品，以防其状况恶化并导致紧急医疗情况；
- 和他待在一起，或确保有人陪他；
- 注意他及其周围环境，以防止其绊倒或跌倒；
- 让他远离机器和危险物品；
- 考虑在他清醒后告知其醉酒时的状态。

## 与醉酒者交谈

以礼貌的方式与对方交谈，使用简单、清晰、正面的语言（如“保持冷静”），不要用负面语言（如“不要打架”），因为他可能会对负面语言反应过度。不要嘲讽、取笑或激怒他。此时不要试图与他郑重地讨论其饮酒行为。

## 送醉酒者回家

你应该阻止对方开车或骑车，如告诉他这样做会对他自己以及其他人造成危险，并为他安排一种安全的交通方式回家。如果不能安全地阻止他开车，你应该打电话报警。

如果可能，你应该把他交给他的家人。如果他在饮酒后独自回家，你应该通过电话确认他是否已安全到家。

## 若醉酒者变得有攻击性怎么办

如果对方变得有攻击性，你应该：

- 保持镇定；
- 尽量缓和局面；
- 用温和、体贴的语气，缓慢、自信地说话；
- 观察他是否有攻击性越来越强的迹象；
- 避免以敌对或威胁的方式与他交谈，
- 避免与他争论；
- 考虑暂停谈话，以便他有机会冷静下来。

任何时候都应该确保自身安全。如果发生暴力事件，你应该寻求适当的紧急援助。

## 寻求医疗救助

如有需要，你勿需害怕为对方寻求医疗救助，即使这样做可能会使醉酒者对其行为负法律责任。同时，你也应意识到，由朋友或家人陪同对方去医院会更好，因为他们可以提供必要的信息。

## 应对酒精中毒相关紧急情况的一般原则

你要了解酒精可以掩盖受伤导致的疼痛。你应该观察对方的呼吸道、呼吸和血液循环。如果他突然停止呼吸，你需要立刻对其实施人工呼吸。如果他出现停搏现象，你则需要立刻对其实施心肺复苏。在把他置于复苏体位前，你应先检查地上是否有碎玻璃。注意给他保暖，以防其体温过低。要意识到一个问题：即使对方身体可能是温暖的，但其体温实际上可能正在下降。

## 当醉酒者呕吐时该怎么办

如果对方呕吐，你应在必要时清理其呼吸道。如果他呕吐但意识清醒，你应让其保持坐姿或将其置于复苏体位。如果他呕吐且神志不清，你应将其置于复苏体位，并陪伴其直到医疗救助到来。如果听任其仰卧，他可能会因呕吐物或舌头阻塞呼吸道而窒息。如果他持续呕吐，你则应呼叫救护车。

## 当醉酒者昏睡时该怎么办

当醉酒者昏睡时，你应该通知其家人。如果很难唤醒对方，你应将其置于复苏体位。如果唤不醒他，你则应呼叫救护车并告知专业救护人员他昏睡前的状况，如喝了多少酒以及正在服用哪些药物等信息。

## 如果酒精中毒导致危险怎么办

你应该明白酒精中毒意味着什么，并且能够识别酒精中毒的症状。如果你怀疑对方酒精中毒，应确保他不是一人独处，也不要给他食物以免窒息。如果担心他已达酒精中毒程度，应立刻送他去医院急诊，不要等待所有酒精中毒症状都出现才寻求医疗救助。

如救助对象出现以下情况，你应该呼叫救护车：

- 呼吸不规律、较浅或较慢；
- 脉搏不规律、微弱或较慢；
- 皮肤是冷的、湿的、颜色苍白或发紫；
- 开始神志不清。

| **酒精中毒**是指血液中的酒精含量过高，可导致死亡。导致酒精中毒的酒精摄入量因人而异。  如果对方出现以下任何症状，则有可能是酒精中毒：   - 呼吸不规律、浅或慢 - 脉搏不规律、微弱或慢 - 皮肤冰冷、湿粘或发紫 - 意识不清乃至昏迷 |
| --- |

## 其他酒精相关紧急事件

如果出现饮酒相关紧急事件，你应该告知对方家人。如果他出现以下情况，你应该寻求医疗救助或致电紧急服务：

- 出现头部受伤的迹象（如呕吐且说话语无伦次）；
- 表达出想要自杀的想法（如他说想自杀）或表现出自杀行为（如想要走到马路中间）；
- 酒中疑似被下药。

## 酒精戒断

你要明白酒精戒断的含义，并能够识别酒精戒断的症状。你也要了解，在不服药的情况下戒酒可能会导致癫痫发作。

如对方出现以下情况，你应该寻求医疗救助：

- 表现出严重的酒精戒断症状；
- 不再酗酒，但变得神志不清、糊涂或产生幻觉；
- 长期大量饮酒但突然决定戒酒。

如果对方戒断症状严重，有生命危险，且不能及时获得有效专业救助，你可以让他少量饮酒以暂时缓解症状。

| **酒精戒断**是指一个经常大量饮酒的人在戒酒后或喝的比平时少得多时可能出现的一系列症状，包括：   - 自主神经活动亢进（例如，出汗或脉搏超过100次/分钟） - 手部震颤加重 - 失眠 - 恶心或呕吐 - 短暂性的视、触或听幻觉或错觉 - 精神运动性激越 - 焦虑 - 癫痫大发作   *摘自美国精神医学学会（2013）精神障碍诊断与统计手册（DSM-5）* |
| --- |
